# Primary care patient willingness for genetic testing for salt-sensitive hypertension: a cross sectional study

**DOI:** 10.1186/1471-2296-14-149

**Published:** 2013-10-09

**Authors:** Masanobu Okayama, Taro Takeshima, Ryusuke Ae, Masanori Harada, Eiji Kajii

**Affiliations:** 1 Division of Community and Family Medicine, Center for Community Medicine, Jichi Medical University, Yakushiji 3311-1, Shimotsuke-city, Tochigi 329-0498, Japan; 2 Division of Public Health, Center for Community Medicine, Jichi Medical University, Yakushiji 3311-1, Shimotsuke-city, Tochigi 329-0498, Japan; 3 Department of Support of Rural Medicine, Yamaguchi Grand Medical Center, Ohsaki 77, Hofu, Yamaguchi 747-8511, Japan

**Keywords:** Attitudes, Genetic testing, Salt-sensitive hypertension, Primary care, Outpatient

## Abstract

**Background:**

The current research into single nucleotide polymorphisms has extended the role of genetic testing to the identification of increased risk for common medical conditions. Advances in genetic research may soon necessitate preparation for the role of genetic testing in primary care medicine. This study attempts to determine what proportion of patients would be willing to undergo genetic testing for salt-sensitive hypertension in a primary care setting, and what factors are related to this willingness.

**Methods:**

A cross-sectional study using a self-report questionnaire was conducted among outpatients in primary care clinics and hospitals in Japan. The main characteristics measured were education level, family medical history, personal medical history, concern about hypertension, salt preference, reducing salt intake, and willingness to undergo genetic testing for salt-sensitive hypertension.

**Results:**

Of 1,932 potential participants, 1,457 (75%) responded to the survey. Of the respondents, 726 (50%) indicated a willingness to undergo genetic testing. Factors related to this willingness were being over 50 years old (adjusted odds ratio [ad-OR] = 1.42, 95% Confidence interval = 1.09 – 1.85), having a high level of education (ad-OR: 1.83, 1.38 – 2.42), having a family history of hypertension (ad-OR: 1.36, 1.09 – 1.71), and worrying about hypertension (ad-OR: 2.06, 1.59 – 2.68).

**Conclusions:**

Half of the primary care outpatients surveyed in this study wanted to know their genetic risk for salt-sensitive hypertension. Those who were worried about hypertension or had a family history of hypertension were more likely to be interested in getting tested. These findings suggest that primary care physicians should provide patients with advice on genetic testing, as well as address their anxieties and concerns related to developing hypertension.

## Background

During the past few years, there has been an explosion in genomics; the advent of genome-wide association studies has revealing hundreds of deoxyribonucleic acid (DNA) variants significantly associated with common diseases [1,2]. This advance holds the promise of personalized medicine, with the potential to enhance human health through more effective prevention, diagnosis, and treatment, [3] and direct-to-consumer genetic testing services provided by private companies are rapidly increasing in popularity [4]. Genetic testing is becoming increasingly accessible to anyone who wishes to obtain information about his or her genetic profile.

Genomics research has traditionally focused on rare conditions, such as hereditary diseases. However, the current research into single nucleotide polymorphisms has extended the role of genetic testing to the identification of increased risk for common medical conditions, [2] as well as lifestyle-enhancing information. For example, nutrigenetics uses genetic information to provide personalized diet and lifestyle advice [5]. Many consumers of direct-to-consumer personal genetic testing are expected to seek physician advice to help them make sense of their test results, [6] increasing the need for advice on genetic testing in all branches of medicine—especially primary care [7]. In addition to reproductive risks, adult-onset Mendelian inheritance disorders, and normal genetic variations in drug metabolism, common diseases with multifactorial etiologies, such as cardiovascular disease, diabetes mellitus, and hypertension are categories of genetic medicine that are relevant in primary care [7,8].

To appropriately explain the results of genetic testing, practitioners must better understand patients’ awareness of genetic testing. Most previous studies exploring awareness of genetic testing were conducted using patients with various forms of cancer, such as beast, [9] colorectal, [10] and ovarian cancer [11]. Studies have also focused on patients attending cancer clinics, [12-14] or receiving direct-to-consumer genetic testing [15]. Two studies performed in primary care settings examined awareness of genetic risk of hereditary diseases [16] and breast cancer [17]. However, the proportion of primary care patients who are willing to undergo genetic testing for common diseases, aside from cancer, remains unknown.

Treatment of hypertension is the most common reason for primary care outpatient visits for non-pregnant adults in both the United States [18] and Japan [19]. Excessive salt intake is associated with increased blood pressure, [20] but several gene polymorphisms associated with salt-sensitive hypertension have also been identified [21]. Consequently, it seems desirable to clarify what proportion of patients would be willing to undergo genetic testing for salt-sensitive hypertension, and what factors were related to this willingness in outpatients from a primary care setting. The present study attempts to fill this gap.

## Methods

### Study design

This cross-sectional study used a self-administered questionnaire.

### Participants and measurements

Anonymous questionnaires were distributed to consecutive outpatients over 20 years old who visited the primary care departments of three clinics and two small hospitals in Japan. Data were collected during two-week periods at each clinic or hospital, from September to December 2009. Research assistances handed out the questionnaires to the patients at the reception counter and told that they were not remunerated for participation and could decline to participate without penalty. The patients filled out the questionnaire in the waiting room. Primary care physicians were not informed as to whether patients answered the questionnaire or not. The questionnaire asked about participants’ age, sex, education level, occupation (“Are you a healthcare worker?” [yes/no]), family and personal medical history (with regard to hypertension, diabetes mellitus, stroke, and myocardial infarction), body mass index (BMI), worries about hypertension and diabetes mellitus (“Do you worry about hypertension” and “Do you worry about diabetes mellitus”, respectively), salt preference (“Do you prefer salty foods?” ), current lifestyle behaviors (smoking, drinking, regular exercise, and reducing salt intake), and willingness to undergo genetic testing for salt-sensitive hypertension (“Would you want to undergo genetic testing that detected whether or not you have a genetic risk predisposing you to hypertension by excessive salt intake?” ). We a provided a description of genetic testing for salt-sensitive hypertension, but no any additional information.

### Analysis

Statistical analyses were performed using STATA/SE version 12.1 (Stata Corp LP, College Station, Texas, TX, US). The significance threshold was set at 0.05. Descriptive statistics were mean ± standard deviation (SD) for age, and proportion for all other variables. Participant age was divided into three categories (less than 50 years old, 50–64, and 65 and over) and proportion was calculated for each category. Obesity was defined as having a BMI greater than 25. The Japanese have defined obesity as any BMI greater than 25 [22]. Chi-square tests were used to compare the proportion of patients for each item who were or were not willing to undergo genetic testing.

We conducted logistic regression analyses to determine factors related to willingness to undergo genetic testing. For these analyses, the sample was divided dichotomously on the basis of age into over-50-years old and under-50-years groups. On the basis of the patients’ answers regarding education level, persons who had graduated college and university were classified as the high education group, while all others were classified as the non-high education group. Using univariate analysis, crude odds ratios (ORs) and 95% confidence intervals (CIs) were calculated for age, sex, education level, occupation, family medical history, personal medical history, obesity, worries about hypertension and diabetes mellitus, salt preference, and four current lifestyle behaviors. Adjusted ORs (95% CI) were then obtained, adjusting for variables that were significantly related in the univariate analyses.

### Ethics

Approval was obtained for the study protocol and questionnaire from the Jichi Medical University Review Board.

## Results

Of the 1,932 outpatients who visited the study sites during the study period, 1,457 (75% response rate) completed the survey (males: 552; 38%). Mean age of participants was 58.1 ± 17.3 years, and 726 (50%) were willing to undergo genetic testing for salt-sensitive hypertension (Table 1).

**Table 1:** Demographic characteristics (N = 1,457)

	***n *(%)**
Age in years (mean ± SD)	(58.1 ± 17.3)
<50	469 (32)
50-64	406 (28)
65+	582 (40)
Sex	
Male	552 (38)
Female	905 (62)
Occupation	
Healthcare worker	98 (7)
Not a healthcare worker	1,359 (93)
Education level	
Elementary school	102 (7)
Junior high school	393 (27)
High school	575 (40)
College	291 (20)
University	96 (7)
Family medical history	
Hypertension	550 (38)
Diabetes mellitus	195 (13)
Stroke	256 (18)
Myocardial infarction	120 (8)
Personal medical history	
Hypertension	499 (34)
Diabetes	167 (12)
Stroke	32 (2)
Myocardial infarction	35 (2)
Obesity (BMI > 25)	293 (20)
Worries about	
Hypertension	800 (53)
Diabetes	706 (49)
Salt preference	
Prefers salty foods	869 (60)
Dose not prefer salty foods	588 (40)
Current lifestyle behaviors	
Smoke	241 (17)
Drink	585 (40)
Regularly exercise	578 (40)
Reducing salt intake	796 (55)
Willingness to be tested	726 (50)

*Note:* SD = standard deviation, BMI = body mass index.

Table 2 gives a comparison of the proportions of patients for each item who were and were not interesting in genetic testing. We found no significant differences for salt preference or reducing salt intake between patients who were and were not interesting in genetic testing. Of the participants with and without family histories of hypertension, 43% and 32% were willing to be tested, respectively (chi-square test, p < 0.001). Of the participants who worried or did not worry about hypertension, 64% and 46% were willing to be tested, respectively (chi-square test, p < 0.001).

**Table 2:** Comparison of participants who were and were not willing to undergo genetic testing

	**Willing (*n*** **= 726), *n *(%)**	**Not willing (*n*** **= 731), *n *(%)**	p value *
Age in years			0.004
<50	213 (29)	256 (35)	
50-64	229 (32)	177 (24)	
65+	284 (39)	298 (41)	
Sex			0.446
Male	268 (37)	284 (39)	
Female	458 (63)	447 (62)	
Occupation			0.006
Healthcare worker	62 (9)	36 (5)	
Not a healthcare worker	664 (91)	695 (95)	
Education level			<0.001
Elementary school	37 (5)	65 (9)	
Junior high school	188 (26)	205 (28)	
High school	275 (38)	300 (41)	
College	173 (24)	118 (16)	
University	53 (7)	43 (6)	
Family medical history			
Hypertension	317 (44)	233 (32)	<0.001
Diabetes mellitus	100 (14)	95 (13)	0.663
Stroke	147 (20)	109 (15)	0.007
Myocardial infarction	59 (8)	61 (8)	0.880
Personal medical history			
Hypertension	276 (38)	223 (31)	0.003
Diabetes	85 (12)	82 (11)	0.769
Stroke	20 (3)	12 (2)	0.147
Myocardial infarction	17 (2)	18 (2)	0.880
Obesity (BMI > 25)	153 (21)	140 (19)	0.360
Worries about			
Hypertension	468 (64)	332 (45)	<0.001
Diabetes	380 (52)	326 (45)	0.003
Salt preference			0.109
Prefers salty foods	448 (62)	421 (57)	
Dose not prefer salty foods	278 (38)	310 (43)	
Current lifestyle behavior			
Smoke	108 (15)	133 (18)	0.088
Drink	296 (41)	289 (40)	0.630
Regularly exercise	285 (39)	293 (40)	0.747
Cutting down on salt intake	406 (56)	390 (53)	0.324

*Note:* CI = confidence interval, BMI = body mass index. *chi-square test.

Univariate logistic regression analysis (Table 3) showed that the factors related to participants’ willingness to undergo genetic testing were being over 50 years old (crude OR: 1.30, 95% CI: 1.04–1.62), being a healthcare worker (1.80, 1.18–2.76), being in the high education group (1.60, 1.26–2.02), having a family history of hypertension (1.66, 1.34–2.05) or stroke (1.45, 1.10–1.90), having a personal medical history of hypertension (1.40, 1.12–1.74), and worrying about hypertension (2.18, 1.77–2.69) or diabetes mellitus (1.36, 1.11–1.68). Multivariate logistic regression analysis identified being over 50 years old (adjusted OR [ad-OR]: 1.42, 95% CI: 1.09–1.85), being in the high education group (ad-OR: 1.83, 1.38–2.42), having a family history of hypertension (ad-OR: 1.36, 1.09–1.71), and worrying about hypertension (ad-OR: 2.06, 1.59–2.68) as independently related to willingness to undergo genetic testing.

**Table 3:** Factors related to participant willingness to undergo genetic testing

	**Crude OR (95% CI)**	**Adjusted OR (95% CI)**
Age: ≥50 years	1.30 (1.04–1.62)	1.42 (1.09–1.85)
Sex: male	0.92 (0.75–1.14)	
Occupation		
Healthcare worker	1.80 (1.18–2.76)	1.34 (0.83–2.17)
Education level		
High education group	1.60 (1.26–2.02)	1.83 (1.38–2.42)
Family medical history		
Hypertension	1.66 (1.34–2.05)	1.36 (1.09–1.71)
Diabetes mellitus	1.07 (0.79–1.45)	-
Stroke	1.45 (1.10–1.90)	1.24 (0.93–1.66)
Myocardial infarction	0.97 (0.67–1.41)	-
Personal medical history		
Hypertension	1.40 (1.12–1.74)	0.96 (0.73–1.25)
Diabetes	1.05 (0.76–1.45)	-
Stroke	1.70 (0.82–3.50)	-
Myocardial infarction	0.95 (0.49–1.86)	-
Obesity (BMI > 25)	1.13 (0.87–1.46)	-
Worry about		
Hypertension	2.18 (1.77–2.69)	2.06 (1.59–2.68)
Diabetes	1.36 (1.11–1.68)	1.02 (0.80–1.29)
Salt preference	1.19 (0.96–1.46)	-
Current lifestyle behavior		
Smoke	0.79 (0.60–1.04)	-
Drink	1.05 (0.85–1.30)	-
Regularly exercise	0.97 (0.78–1.19)	-
Cutting down on salt intake	1.11 (0.90–1.36)	-

*Note:* CI = confidence interval, BMI = body mass index.

## Discussion

This study answered the question of what proportion of outpatients visiting primary care clinics and hospitals are willing to undergo genetic testing for salt-sensitive hypertension, and what factors are related to this willingness. This study found that exactly half of the patients in a primary care setting wanted to have their genetic risk of salt sensitivity hypertension assessed. This is consistent with the 43–76% rate at which participants in other studies, including cancer patients, the relatives of cancer patients, and attendees of the educational component of a breast cancer awareness campaign, have been interested in genetic testing for cancers and hereditary diseases in previous studies [9,12,13,16,17].

Half of patients with chronic diseases consider their primary care physician to be the preferred source of information about the role of genetics in their health [6]. However, the vast majority of single nucleotide polymorphisms are associated with very low odds ratios for common diseases [23]. Furthermore, genetic polymorphisms related to salt-sensitive hypertension are not a major contributor to increased blood pressure [24,25]. Although genetic testing to predict common diseases may not yet be available or practical in a primary care setting, the findings in this study indicate that, in the near future, primary care physicians are likely to find that genetic testing and providing advice on genetic testing results are playing a role in their day-to-day practice.

In the present study, no relationships were found between participants’ salt preference or reducing salt intake and their willingness to undergo genetic testing for salt-sensitive hypertension. However, genetic risk information can improve information about nutrition for optimal personal health; [26] in other words, knowing whether one had a salt sensitivity for hypertension or not could contribute to patients’ decisions to modify their salt intake or maintain current eating habits. We were surprised that, in the present study, participants’ preference for a salty taste was in no way related to their willingness to be tested for genetic risk. The findings in this study suggest that disclosing the genetic testing results regarding salt-sensitive hypertension may have little influence on behavioral modification of salt intake. Therefore, future studies should clarify the effects of genetic information regarding risk of salt sensitive hypertension on behavioral changes. In this way, nutrigenetics concerning salt intake could be applied to the primary care setting.

Findings from the present study indicate that age (over 50), a high level of education, a family history of hypertension, and worrying about hypertension were independent factors positively related to willingness to undergo genetic testing for salt-sensitive hypertension. These factors were consistent with previous studies on willingness to be tested for genetic risk of cancer and other hereditary disease [11-13,16,17]. On the basis of these findings, it appears that, regardless of the kind of disease in question, the aforementioned factors might related to general willingness to be tested for genetic risk.

Behavioral modification is more efficient in preventing hypertension in people younger than 50 than in people over 50. However, the findings in the present study indicate that people over 50 years old are more willing than their younger counterparts to undergo genetic testing for salt-sensitive hypertension. Although the positive effects of knowing one’s genetic risk profile on making positive behavioral changes has been established, this benefit can, of course, not be achieved without first undergoing genetic testing. Therefore, improved motivation among patients to know more about their genetic risk of salt-sensitive hypertension can translate into clinically meaningful, cost-effective changes in outcomes for hypertensive patients. More positive changes can be made by finding ways to encourage patients younger than 50 years old to agree to undergo genetic testing for their risk of salt-sensitive hypertension.

Of course, genetic testing has a potential for causing anxiety and distress, especially if a patient learns that he or she is at increased risk for a serious illness. This is exacerbated by the fact that patients expect genetic testing to precisely predict whether they will develop a specific disease, and that genetic testing will improve their ability to manage this disease; they are often unable to grasp the uncertain nature of the information currently available from genetic testing [27,28]. However, a test result— positive and negative—can bring relief from uncertainty and help patients to make informed medical and lifestyle decisions, including taking steps to improve their health-seeking behaviors [29,30]. There is still significant disagreement as to whether genetic testing is overall anxiety causing or reducing, and whether it would be therefore indicated for all consumers. Further, Two studies have reported that information from direct-to-consumer genetic testing causes no additional anxiety to consumers [31,32]. However, a different study reported that testing positive for increased genetic risk of illness was associated with high levels of anxiety and depression [33]. Another study warned that heightened, unnecessary anxiety was a potential negative consequence of genetic testing [34]. While there is still disagreement over the larger issue of genetic testing, the present finding about the role of worrying about hypertension indicates that when disclosing the results of genetic testing, primary care physicians should keep in mind the potential for patients to feel anxious and concerned about developing hypertension, and to offer advice and explanations along with test results. For example, when discussing the results of genetic testing, primary care physicians should be specific, rather than general.

Despite its contributions, this study has several limitations. First, we assessed participant interest in genetic testing without taking into account the factors of cost and level of understanding of genetic testing, simply providing a description of genetic testing for salt-sensitive hypertension. Second, preference for salty foods was assessed using self-report; we did not measure actual daily salt intake. However, patient preference for salty foods does not always correlate with salt intake [35]. Future studies should explore the relationship between actual daily salt intake and willingness to be tested for genetic risk of salt-sensitive hypertension. Third, this study used self-report data, and self-reporting frequently results in considerable error in data presentation. In addition, patients who were highly educated may perform different healthy behaviors from those they reported. These methodological weaknesses might have influenced findings regarding the relationships between salt preference, current lifestyle behaviors, and the willingness to undergo genetic testing. Fourth, although study findings were consistent with those obtained previously in samples using patients of different nationalities, the participants in this study were all Japanese, and cultural, dietary, and genetic differences might have diminished or reinforced the applicability of the findings in this study to other countries. Finally, this study did not assess perceived benefits of genetic testing, such as in predicting the development of hypertension or improving the management of hypertension. This issue possibly influences the proportion of patients who are willing to undergo genetic testing.

## Conclusions

With the rapid advances in genetics research, patients will increasingly have the option to undergo genetic testing for common diseases. In the near future, this may mean that primary care physicians will be required to prepare to advise patients on questions of genetic risk concerning common diseases such as hypertension. Physicians will need to be informed about what information would be most helpful to their patients, what patients are more or less likely to be willing to undergo genetic testing, and how patients will use this information to shape health-promoting behaviors.

## Abbreviations

BMI: Body mass index; CI: Confidence interval; DNA: Deoxyribonucleic acid; OR: Odds ratio; SD: Standard deviation.

## Competing interests

The authors declare that they have no competing interests.

## Authors’ contributions

MO contributed to conception and design, acquisition of data, analysis and interpretation of data, and writing and revision of the manuscript. TT contributed to conception and design and analysis and interpretation of data. RA and MH contributed to acquisition, analysis, and interpretation of data. EK contributed to conception and design and writing and revision of the manuscript. All authors approved the final version of the manuscript to be published.

## Pre-publication history

The pre-publication history for this paper can be accessed here:

http://www.biomedcentral.com/1471-2296/14/149/prepub
